# Characterizing geospatial access to comprehensive breast cancer services in Northern Tanzania

**DOI:** 10.3389/fonc.2026.1952705

**Published:** 2026-10-23

**Authors:** Tara M. Friebel-Klingner, Anne F. Rositch, Hari S. Iyer, Rachel B. Wagner, Emma Joo, Matogoro Kirahi, Nestory Masalu, Racquel E. Kohler

**Affiliations:** 1 Rutgers Global Health Institute, New Brunswick, NJ, United States; 2 Hologic Inc., San Diego, CA, United States; 3 Department of Epidemiology, Johns Hopkins Bloomberg School of Public Health, Baltimore, MD, United States; 4 Rutgers Cancer Institute, New Brunswick, NJ, United States; 5 Emory University, Atlanta, GA, United States; 6 Bugando Medical Center, Mwanza, Tanzania

**Keywords:** access to care, breast cancer, geospatial analysis, sub-Saharan Africa, women’s health

## Abstract

**Background:**

Tanzania has a high breast cancer mortality burden attributed in part to poor access to comprehensive cancer services. The objective of this study was to characterize geospatial patterns of breast cancer patients presenting to the only tertiary hospital in northern Tanzania that provides comprehensive breast cancer care.

**Methods:**

This geospatial analysis used baseline data from adult breast cancer patients diagnosed at Bugando Medical Center (BMC) in Mwanza, Tanzania, between July 2019 and December 2023. Participants were geocoded to their residential subdistricts as defined by the United States Census Bureau. Forty-five subdistricts in the catchment area were identified as the spatial unit of analysis. Age-standardized observed rates depicted the number of patients diagnosed with breast cancer per subdistrict. Global and local Moran’s I statistics assessed spatial autocorrelation of observed breast cancer rates. Ordinary least squares regression models evaluated community-level factors, including population density, the presence of hospitals, and distance to comprehensive cancer care.

**Results:**

We included 379 women diagnosed with breast cancer from the catchment area, with a median of 3 breast cancer cases per subdistrict (IQR: 0-10). Participant ages ranged from 21–90 years old. The mean age-standardized observed breast cancer rate was 4.2 per 100,000 women (IQR: 0-10.1). Observed breast cancer rates were spatially clustered across the entire catchment area (Moran’s Index= 0.289; p=0.001). Additionally, specific subdistricts where clustering and dispersion existed were identified. Ordinary least squares regression identified community-level factors (low population density; p=0.003, greater distance to BMC; p<0.001) associated with lower breast cancer rates.

**Conclusions:**

We identified spatial clustering of breast cancer rates in the Northern region of Tanzania. Spatial clustering aligned with distance to comprehensive cancer care and population density, suggesting that low observed rates in remote areas may reflect underdiagnosis due to diagnostic availability rather than lower disease burden. Decentralizing diagnostic capacity, particularly in low-rate spatial clusters, may enhance equitable access to care for patients facing geographic barriers and improve breast cancer early detection and outcomes.

## Introduction

Breast cancer is the most prevalent cancer affecting women worldwide (1). Among women in sub-Saharan Africa (SSA), breast cancer is the most frequently diagnosed and the leading cause of cancer-related mortality (2). Incidence in many SSA countries is projected to increase substantially (85.7%) by 2040 (3), with overall burden expected to continue rising through 2050 (4). Limited access to services contributes to late-stage diagnosis in resource-constrained settings (5–7) and five-year survival remains low at approximately 40% (8).

Multiple barriers across SSA contribute to delayed diagnosis, treatment initiation and abandonment (9, 10), which are compounded by suboptimal quality of breast cancer care across the continuum (11). Patient access to breast cancer services is constrained by limited diagnostic infrastructure and capacity, high out-of-pocket costs associated with obtaining care, and fragmented care pathways (12, 13). At the health-system level, cancer care is restricted by pathology personnel shortages, equipment failures, and lack of immunohistochemistry (IHC) (14–16). Regarding treatment, drug stockouts and centralized oncology services limit the availability of and access to essential cancer medicines and therapies closer to where patients live (13, 17–19). At the community level, rural residence has been associated with poorer survival (20), whereas living within communities with cancer referral hospitals has been associated with shorter diagnostic delays (21). Together, these challenges exacerbate breast cancer disparities.

Like many other low-resource countries in SSA, women in Tanzania experience poor breast cancer outcomes. Interruptions in treatment adherence and completion, late-stage diagnosis, and low five-year survival reflect ongoing resource constraints (22–24). Though 90% of Tanzania’s population lives within 5km of a health facility, specialized oncology services remain concentrated in urban areas (25). As a result, travel times increase considerably for greater levels of care, from three to six hours for residents of sparsely populated areas (26, 27). These geographic disparities highlight the need for spatially informed strategies to improve access to breast cancer care services.

Geospatial analysis using geographic information systems (GIS) integrates spatial data from multiple sources to examine how geographic location influences engagement in care by identifying areas of limited access (28–30). Previous studies in SSA have demonstrated that spatial analysis can identify spatial clustering of cancer presentation and associations between travel distance and care utilization, revealing potential inequities in access (31, 32). These types of findings can inform targeted interventions and guide allocation of limited resources to improve access to cancer care. Therefore, this study aimed to characterize geographic variation and spatial clustering in observed breast cancer rates for women presenting for breast cancer services at Bugando Medical Center (BMC). Because BMC is the only tertiary facility providing comprehensive breast cancer care in the region, examining the geographic distribution of patients who successfully present for care provides insight into spatial patterns of realized access to comprehensive breast cancer services.

## Materials and methods

### Study setting

We conducted a cross-sectional ecological geospatial analysis of breast cancer rates across 45 subdistricts in the Lake Zone region of Tanzania. Public health care in Tanzania includes primary (district), secondary (regional referral), tertiary (zonal and national) and specialized hospitals. The health system generally follows a progressive cascade where patients first access care at lower-level facilities close to home and are then referred to higher-level tertiary hospitals, where cancer diagnostic and comprehensive treatment services are primarily available. In the highly populated Northern Lake Region, Mwanza, Bugando Medical Center (BMC) serves as the only tertiary referral hospital, providing cancer services to over twenty million residents across a large geographical region. Its catchment area spans approximately 200 kilometers (km) to the south, north, and west, and is over 1,000 km away from the nearest other tertiary hospital.

### Data sources and measures

Observed breast cancer rates per 100,000 population (women aged ≥20 years) were calculated and age-standardized to Tanzania’s overall population for each administrative unit in BMC’s catchment area. Women with breast concerns presenting to BMC and subsequently diagnosed with breast cancer between July 2019 and December 2023 were included. Data were extracted from BMC hospital registries and electronic medical records. Patient-level data included age, insurance status, pathology information, loss to follow up, and subdistrict of residence. Cases were geocoded to their residential corresponding United States Census Bureau (USCB)-defined subdistrict (33).

Community-level covariates included travel distance to care (km), population density, and presence or absence of district or regional hospitals. These variables were selected *a priori* to capture geographic access to diagnostic services. Travel distance was calculated as distance in kilometers (km) traveled from the centroid of each subdistrict to BMC (34), determined using Google Maps (Google LLC, Mountain View, CA, USA) (35). Population density was calculated using the population relative to the polygon area from a publicly available dataset (33). We restricted analysis to the presence or absence of higher-level facilities (i.e., district hospitals, regional referral hospital, referral hospitals).

### Geospatial and statistical analysis

Administrative subdistricts in Tanzania were selected as the unit of analysis. Spatial boundary data were obtained from a publicly available shapefile provided by the USCB, which outlines 52 subdistricts within the BMC catchment area. One subdistrict, Ukerewe, was excluded from the analysis because it is an island and does not share borders with any other subdistrict. Medical records did not distinguish between subdistricts and their corresponding municipalities (e.g., “Bukoma” was recorded without differentiation between Bukoma subdistrict and Bukoma Municipality). Hence, to ensure consistency between geographic boundaries and medical record data, subdistricts that also had municipalities were aggregated to align with medical record data. Similarly, because these municipalities were located within or adjacent to the subdistricts, they were dissolved into single subdistrict-level polygons. Thus, our final analytic data set consisted of 45 subdistricts.

Choropleth maps created in ArcGIS Pro (Esri, Redlands, CA) depicted the age-standardized observed breast cancer rates by subdistrict. Global spatial clustering of breast cancer rates was assessed using Moran’s I statistic. Spatial relationships were defined using a first-order queen contiguity matrix (shared edges or corners) with row standardization. Statistical significance was evaluated using 999 permutation tests (36–38).

Local indicators of spatial association (LISA) were estimated using Anselin Local Moran’s I to identify clusters and spatial outliers. Statistically significant clusters and outliers were identified at the 95% confidence level. Subdistricts were classified as high-high (HH) if they had high observed breast cancer rates and were surrounded by neighboring subdistricts with similarly high rates, and as low-low (LL) if they had low observed breast cancer rates and were surrounded by neighboring subdistricts with similarly low rates. High-low (HL) and low-high (LH) subdistricts were classified as spatial outliers when their observed breast cancer rates differed from those of neighboring subdistricts. Statistical significance was determined using permutation-based p-values.

To further evaluate the drivers of spatial clustering, we explored community-level characteristics across LISA cluster types. Mean values of distance to care, population density, and health facility measures were calculated for HH, LL, HL, LH, and non-significant areas. Differences across groups were assessed descriptively, using analysis of variance (ANOVA), and chi-square tests as appropriate.

Statistical analysis were performed using STATA version 19.5 (College Station, TX) (39). Multivariable ordinary least squares (OLS) regression was used to examine associations between observed breast cancer rates and community-level covariates. We assessed potential non-linear relationships by adding quadratic terms for community level variables. Residual spatial autocorrelation was assessed using Moran’s I applied to model residuals. Results are reported as unadjusted and adjusted coefficients with corresponding 95% confidence intervals, and statistical significance was defined using a two-sided α of 0.05.

## Results

### Sample characteristics

Between July 2019 and December 2023, 379 women presented to BMC with breast concerns from the catchment area and were subsequently diagnosed with breast cancer (**Table 1**). Patients had a median age of 50.0 (IQR:40, 60), 46% had private insurance, 52.2% completed pathological staging, of which 65% were diagnosed with late-stage disease (i.e., stage III and IV). Patients traveled a median distance of 155 km (IQR: 18, 274); 47.8% were lost to follow-up, 52.2% lived in a subdistrict with a regional hospital, and 67.3% lived in a subdistrict with a district hospital.

**Table 1:** Characteristics of the breast cancer cohort.

Demographics	Data Value N (%)
Total sample	379 (100%)
Age (years)* Median (IQR)	50.0 (40.0, 60.0)
Distance to BMC*	
(Kilometers) Median (IQR)	149.0 (18, 274)
Insurance [n, (%)]	
No	205 (54.1%)
Yes	174 (45.9%)
Completed pathology staging [n, (%)]	
No	181(47.8%)
Yes	198 (52.2%)
Among those with pathology [n, (%)]	
Early Stage (I/II)	70 (35.4%)
Late Stage (III/IV)	128 (64.7%)
Lost to follow-up [n, (%)]	
No	198 (52.2%)
Yes	181 (47.8%)
Live in a subdistrict with a regional hospital [n, (%)]	
No	181 (47.8%)
Yes	198 (52.2%)
Live in a subdistrict with a district hospital [n, (%)]	
No	124 (32.7%)
Yes	255 (67.3%)

Categorical variables are reported as n, frequency and % proportion; *continuous variables are reported as medians with interquartile range (IQR).

The cancer cases resided in 32 of the 45 analytic subdistricts. The median number of breast cancer cases per subdistrict was 3 (IQR: 0-10). The median age-standardized rate was 4.2 per 100,000 women (IQR: 0-10.1). Observed breast cancer rates varied substantially across administrative units, with clear geographic heterogeneity observed on choropleth map (**Figure 1**). Age adjusted breast cancer rates per subdistrict are shown in **Supplementary Table 1**.

**Figure 1:**
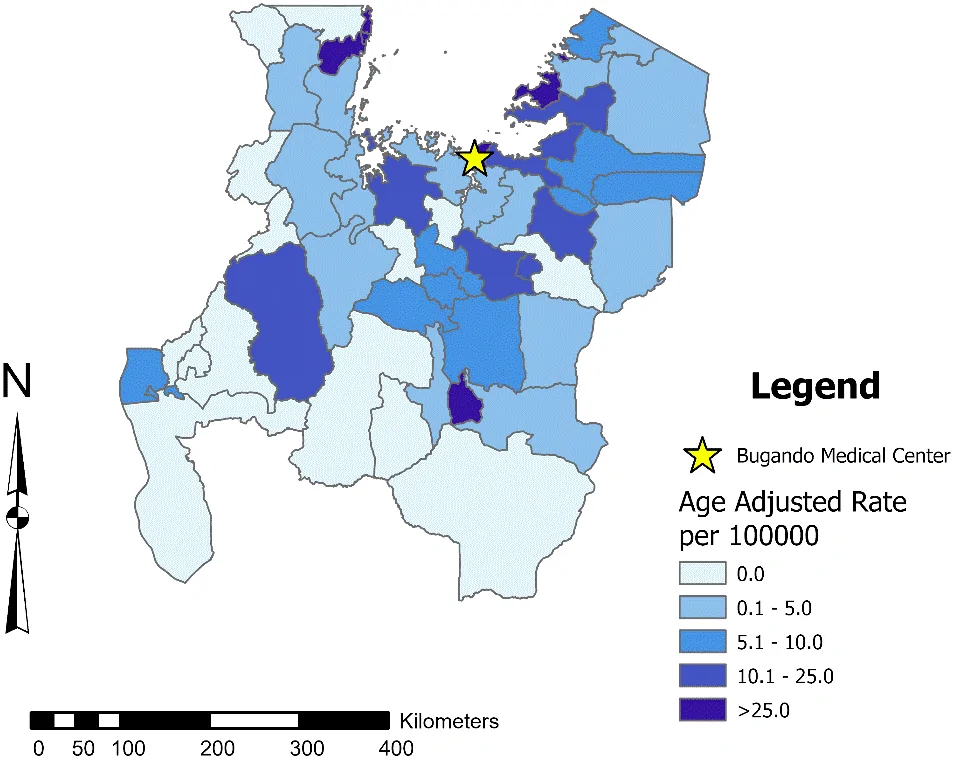
Observed breast cancer rates per subdistrict in the BMC catchment area.

### Global spatial clustering

Global Moran’s I analysis demonstrated significant spatial clustering of breast cancer rates (Moran’s I = 0.289, p = 0.001), indicating that rates were not randomly distributed but instead exhibited geographic dependence.

### Local spatial clustering

LISA analysis identified distinct spatial clusters and outliers of breast cancer rates (**Figure 2**). High–high clusters were observed closest to BMC in Ilemela, Magu, and Nyamagana. These clusters represented areas with elevated rates (Ilemela rate: 67.8 per 100,000; Magu rate: 18.0 per 100,000, and Nyamagana rate: 47.7 per 100,000) surrounded by neighboring high-rate regions. Low–low clusters were identified in Biharamulo (rate: 2.7 per 100,000) and Bukombe (4.7 per 100,000), indicating areas of consistently low observed rates. One high-low outlier was identified in Kibondo (rate: 13.6 per 100,000).

**Figure 2:**
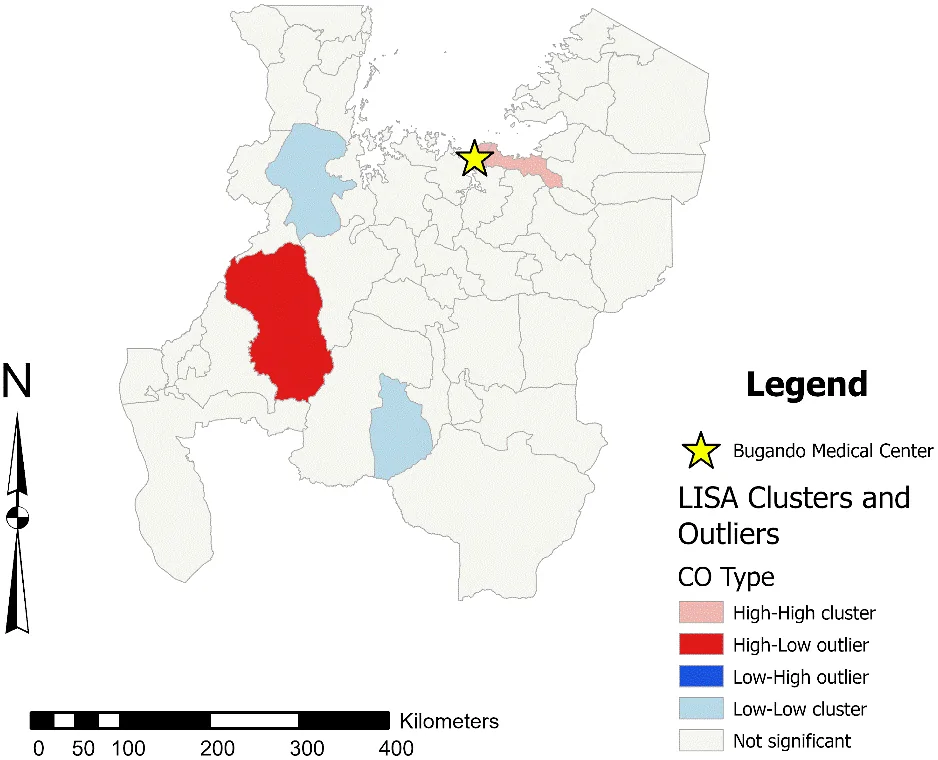
LISA clusters and outliers of observed breast cancer rates.

### Cluster characteristics

Community-level characteristics differed across LISA cluster types (**Table 2**), specifically travel distance (p<0.01) and population density (p<0.03). High–high clusters were generally located in areas with higher population density (840 persons/km²) and shorter distances to care (27.7km), whereas low–low clusters were located further from BMC (260 km) with lower population density (26.8 persons/km²) and reduced access to health services (no regional hospitals). When we examined patient-level characteristics (age, insurance status, pathology, and lost to follow-up) by cluster type, we did not observe any significant differences (results not shown).

**Table 2:** Community-level characteristics by clustered and outlier subdistricts.

Cluster/outlier	All	Not significant	HH	HL	LL
Sub-districts (n)	45	39	3	1	2
BC Cases (n)`	379	228	115	32	5
BC PR Rate; mean (SD)	4.0 (5.5)	3.3 (4.5)	16.4 (8.7)	5.5 (.)	1.4 (0.5)
Distance (km)**; mean (SD)	283.3 (158.9)	303.6 (153.4)	27.7 (31.6)	365.0 (.)	260.5 (6.4)
Population density*; mean (SD)	290.2 (749.5)	275.1 (776.1)	840.0 (713.5)	12.3 (.)	26.8 (16.9)
District hospital [n, (%)]					
No	10 (22.2%)	9 (23.1%)	1 (33.3%)	0 (0.0%)	0 (0.0%)
Yes	35 (77.8%)	30 (76.9%)	2 (66.7%)	1 (100.0)	2 (100.0%)
Regional hospital [n, (%)]					
No	31 (68.8%)	27 (69.2%)	2 (66.7%)	1 (100.0)	2 (100.0%)
Yes	14 (31.1%)	12 (30.8%)	1 (33.3%)	0 (0.0%)	0 (0.0%)

HH, high-high; HL, high-low; LL, low-low; **distance, p<0.001; *population density p<0.03; ANOVA test.

### Regression analysis

In multivariable OLS models, breast cancer rates were associated with distance to care and population density (**Table 3**). Quadratic terms were assessed but not significant (results not shown). Areas farther from care had lower observed rates. For every 1 km increase in distance, breast cancer rates decreased by 0.04 per 100,000 (p<0.001). Higher population density was associated with higher rates (β = 0.008, p < 0.003), indicating that more densely populated areas have higher detected rates. Measures of hospital availability were not significantly associated with breast cancer rates in the adjusted analysis.

**Table 3:** Results of OLS regression with spatial weights matrix for community level factors.

OLS regression	Unadjusted coefficient	95% CI	p-value	Adjusted Coefficient	95% CI	p-value
Distance	-0.04	-0.06, -0.01	<0.01	-0.04	-0.06, -0.02	<0.01
Population density	0.01	<0.01, 0.01	<0.01	0.01	<0.01, 0.01	<0.01
Regional hospital	13.31	4.12, 22.46	0.01	5.40	-3.76, 14.60	0.24
District hospital	-5.60	-15.50, 4.24	0.26	-0.94	-8.66, 6.79	0.81

### Residual spatial autocorrelation

After adjustment for community-level covariates, residual spatial autocorrelation was no longer statistically significant (Moran’s I = 0.116, p = 0.104). Visual inspection of residual maps demonstrated reduced clustering compared to the unadjusted rates (**Supplementary Figure 2**). Absence of statistically significant residual spatial autocorrelation indicates that the included covariates explained the observed spatial clustering.

## Discussion

Observed breast cancer rates in this study demonstrated significant spatial clustering across the BMC catchment area. The identified high- and low-rate clusters highlight geographic variation in breast cancer presentation and may reflect inequities in access to diagnostic services. These patterns may be influenced by community-level factors, including distance to care and population density. Taken together, these findings may help guide targeted allocation of resources to underserved areas where strengthening diagnostic pathways could have the greatest impact.

In this study, although the Global Moran’s I confirmed spatial clustering, the lack of spatial autocorrelation in OLS residuals implies that covariates capture this geographic variation (population density and travel distance). Observed breast cancer rates were lower for areas further from the tertiary hospital, which could potentially reflect reduced detection instead of lower disease burden, consistent with previous evidence of geographic disparities in screening across SSA (40). LISA analysis identified discrete high and low observed rate clusters and one outlier, with high-rate clusters closest to BMC, suggesting that spatial clustering of breast cancer rates could be influenced by proximity to diagnostic infrastructure (41). Kibondo emerged as a high-low spatial outlier, potentially reflecting localized differences in access to care (e.g., referral pathways, transportation, non-governmental organizations) compared with surrounding subdistricts, as another Tanzanian study documented (40, 42).

We found that travel distance was a barrier, whereas population density served as an indirect proxy for care access; hospital counts alone did not adequately account for the observed clustering. Other studies of multi-country geospatial analysis demonstrate regional variation in travel time and distance to oncology care, including disparities in proximity to treatment centers within Uganda and Nigeria (43, 44). Geospatial modeling has further revealed that a large proportion of populations live beyond reasonable travel thresholds for diagnostic services, highlighting persistent geographic inequities in cancer care access (45–47). Additionally, prior studies across SSA have linked greater travel distance to breast cancer care facilities with delayed diagnosis and advanced-stage presentation (47, 48). Rural residence has also been associated with poorer outcomes, such as advanced tumor stage (49).

Consistent with our findings, prior literature also suggests that geographic disparities in outcomes reflect broader structural and health system barriers rather than physical distance alone. For instance, several studies have documented that many SSA hospitals provide only fragmented components of breast cancer care without full diagnostic capacity (46, 50). These gaps often require patients to navigate prolonged diagnostic pathways involving repeated healthcare visits, delayed referrals, and other structural barriers before receiving a formal diagnosis (51–53). Across other African countries, including Tanzania, Nigeria, Ghana, Uganda, and Kenya, transportation costs and financial burden have further contributed to biopsy delays and women’s ability to initiate or continue treatment after being diagnosed (54–56). Indeed, we also observed a high proportion of patients lost to follow-up after being diagnosed, which highlights the challenge of continuity of breast cancer care in this setting and limits the ability to characterize treatment initiation, completion, and outcomes.

We found an overall observed breast cancer rate of 4.2 per 100,000 across the subdistricts. Although this is considerably lower than the country’s estimated incidence rate from Global Cancer Observatory (GLOBOCAN) (35–40 per 100,000 in 2022) (1), we suspect the difference is due to many women not reaching services and remaining undiagnosed. Higher observed breast cancer rates in urban areas or those closer to BMC likely reflect improved diagnostic availability, while low-rate clusters may indicate limited detection in remote areas.

The results of this analysis should be interpreted in light of some limitations, primarily related to the availability and granularity of routinely collected data in SSA. Observed breast cancer rates reflect observed diagnoses rather than underlying population-based incidence rates and are therefore influenced by healthcare utilization and diagnostic availability. No breast cancer cases were observed from 13 subdistricts. In this spatial analysis, the absence of observed cases should not be interpreted as absence of breast cancer in these areas and may reflect differences in referral, access to care, or case ascertainment. Available medical records did not capture patients’ initial points of care and referral pathways prior to BMC presentation, limiting our ability to further characterize these patterns. Similarly, lack of household income and educational attainment data at the subdistrict level limited our ability to account for socioeconomic factors that may influence access to care (57). Exact patient address locations were unavailable, which may have led to measurement error in the estimation of distance from subdistrict centroid to the hospital and attenuated observed associations. As a result, our findings reflect subdistrict spatial patterns of access barriers.

## Conclusion

Geographic barriers to accessing diagnostic care may contribute to high breast cancer mortality in Tanzania. These findings could inform cancer control efforts in the northern region in Tanzania and similar settings. Spatial variation in observed breast cancer rates appears to reflect differences in diagnostic availability rather than the distribution of health facilities alone. These geographic disparities highlight the need for spatially-informed strategies to improve access to comprehensive breast cancer care services, as patients living farther from referral hospitals likely face significant delays in evaluation, leading to lower observed rates that could potentially reflect underdiagnosis. This study highlights how future work to strengthen decentralized diagnostic capacity and improve referral pathways, particularly in low-rate spatial clusters, is needed and may improve equity in early detection and outcomes.

## Data Availability

The raw data supporting the conclusions of this article will be made available by the authors, without undue reservation.

## Supplementary material

Supplementary Figure 1 Moran’s I Scatterplot.

Supplementary Figure 2 Residual spatial autocorrelation.

Supplementary Table 1 Observed breast cancer rates per subdistrict in the BMC Catchment Area.
