# Supplementary material for: Characterizing geospatial access to comprehensive breast cancer services in Northern Tanzania
Source: Front Oncol. 2026 Oct 23;16:1952705. doi: 10.3389/fonc.2026.1952705 (PMC13739489; doi:10.3389/fonc.2026.1952705)
Supplement: Supplementary file 1 [file SupplementaryFile1.docx]

**Supplementary Materials**

**Supplementary eTable 1. Observed Breast Cancer Rates per Subdistrict in the BMC Catchment Area**

| **Subdistrict** | **Cases** | **Female Population >20 Years** | **Unadjusted Rate** | **Age Adjusted Rate** | **Cluster Type** |
| --- | --- | --- | --- | --- | --- |
| Bariadi | 10 | 105822 | 9.45 | 9.60 |  |
| **Biharamulo** | **3** | **109263** | **2.75** | **2.71** | **LL** |
| Buhigwe | 0 | 74722 | -- | 0 |  |
| Bukoba/Bukoba Municipal | 33 | 127012 | 25.98 | 25.74 |  |
| **Bukombe** | **2** | **46946** | **4.26** | **4.74** | **LL** |
| Bunda | 10 | 92212 | 10.84 | 10.09 |  |
| Busega | 8 | 52176 | 15.33 | 14.11 |  |
| Butiama | 3 | 63515 | 4.72 | 4.16 |  |
| Chato | 1 | 97680 | 1.02 | 1.25 |  |
| Geita | 23 | 209455 | 10.98 | 12.98 |  |
| Igunga | 3 | 102820 | 2.92 | 2.95 |  |
| **Ilemela** | **54** | **101763** | **53.06** | **67.82** | **HH** |
| Itilima | 6 | 76332 | 7.86 | 7.85 |  |
| Kahama/Kahama Town | 13 | 204169 | 6.37 | 6.75 |  |
| Kakonko | 0 | 31277 | -- | 0 |  |
| Kaliua | 0 | 119250 | -- | 0 |  |
| Karagwe | 5 | 109446 | 4.57 | 4.28 |  |
| Kasulu/Kasulu Town | 0 | 129358 | -- | 0 |  |
| **Kibondo** | **9** | **67326** | **13.37** | **13.64** | **HL** |
| Kigoma/Kigoma Ujiji Municipal | 10 | 121359 | 8.24 | 7.95 |  |
| Kishapu | 0 | 67195 | -- | 0 |  |
| Kwimba | 3 | 106722 | 2.81 | 2.93 |  |
| Kyerwa | 0 | 92134 | -- | 0 |  |
| **Magu** | **14** | **78403** | **17.86** | **18.00** | **HH** |
| Maswa | 11 | 82874 | 13.27 | 12.67 |  |
| Mbogwe | 0 | 41305 | -- | 0 |  |
| Meatu | 2 | 73049 | 2.74 | 2.95 |  |
| Missenyi | 0 | 60406 | -- | 0 |  |
| Misungwi | 5 | 95256 | 3.13 | 2.95 |  |
| Muleba | 3 | 155956 | 1.92 | 1.90 |  |
| Musoma/Musoma Municipal | 31 | 89063 | 34.81 | 34.22 |  |
| Ngara | 0 | 70116 | -- | 0 |  |
| **Nyamagana** | **47** | **132660** | **35.43** | **47.74** | **HH** |
| Nyang'hwale | 0 | 36297 | -- | 0 |  |
| Nzega | 7 | 131359 | 5.33 | 5.60 |  |
| Rorya | 6 | 72670 | 8.26 | 7.34 |  |
| Sengerema | 8 | 175187 | 4.57 | 4.66 |  |
| Serengeti | 2 | 67309 | 2.97 | 3.06 |  |
| Shinyanga/Shinyanga Municipal | 21 | 130040 | 16.15 | 16.43 |  |
| Sikonge | 0 | 47692 | -- | 0 |  |
| Tabora Municipal | 20 | 61888 | 32.32 | 34.32 |  |
| Tarime | 4 | 85206 | 4.69 | 5.00 |  |
| Urambo | 0 | 52802 | -- | 0 |  |
| Uvinza | 0 | 105908 | -- | 0 |  |
| Uyui | 2 | 108447 | 1.84 | 2.17 |  |
| Notes: HH, high-high; HL, high-low; LL, low-low | | | | | |

**Supplementary eFigure 1. Moran’s I Scatterplot**

**Supplementary eFigure 2. Residual Spatial Autocorrelation**
